# Tape-Worm

**Published:** 1866-10

**Authors:** J. V. Herriott

**Affiliations:** Valparaiso, Ind.


### ARTICLE XXXVII.

### TAPE-WORM.—(*Tænia Solium.*)

BY J. V. HERRIOTT, M.D., Valparaiso, Ind.

The following case of tape-worm tends to strengthen the reputed efficacy of pumpkin seeds (*cucurbita pepo*) in expelling tape-worm from the human intestines:—Geo. G., *æt.* 24, presented himself at my office, June 7th, 1866, complaining of having been "sick for two years," and wished to know, "what is the matter with me?" He had dyspeptic symptoms, with colic pains, especially after eating; had a gnawing, craving sensation for food, the attempt to gratify which, produced satiety and sickness; said he had been passing "worms" for the last two years; had nervous tremors, and was weak; could not endure work from one meal to another, on account of weakness; said he was getting worse all the time, and now could "work but little." Upon his describing the "worms" passed, I diagnosed tape-worm, and requested him to bring me a specimen, which he did, an examination of which confirmed me in the diagnosis I had made.

I directed him to take two ounces of the kernels of pumpkin seeds and make them into an emulsion with sugar and water, and the next morning, fasting, to take all the emulsion at once, and in two hours to take two tablespoonfuls of castor-oil, with one teaspoonful of spirits of turpentine, and to repeat the dose in two hours more, if the first did not operate. The first dose started the worm from his moorings, and three feet were discharged; but he, being anxious to complete the "job," repeated the dose, and in a short time sixteen feet were discharged, which he brought to me at midday, preserved as directed. On careful examination, the head was not found on either end of the worm; I, therefore, directed him to repeat the treatment, which he did, but without further development of worm.

This man said he had passed at least six inches of "worms" every day for the last two years; sometimes dropping down into his boots while at work. The passing of segments of the worm was, no doubt, the means of keeping him up as long as it did, for, if one-half of the length he says he passed had been retained in the intestines, grave symptoms would have been manifested much earlier.

There is one fact more in this case, which has a bearing on the etiology of tape-worm. Having observed that Dr. CABBOLD expressed an opinion before the British Association, "that tape-worm is often contracted from eating beef and veal," I questioned my patient on this point, and elicited the fact that in the fall of the year before he began to pass the segments, (which was in the spring,) he was in the habit of eating raw beef, for which he says he has a strong relish. We further remark, only, that since the expulsion of the worm, this man appears to have new life and energy infused into him. He feels well, eats well, sleeps well, and works well.

---

THE BANEFUL EFFECTS OF NICOTINE PREVENTED.—M. Melsens has found that tobaccos, from various countries, contain nicotine in very different proportion. In tobacco from some parts of France (*e. g.*, the department of the Lot) there is 7.96 per cent. of nicotine; whilst Havana tobacco contains only two per cent. He proposes to smokers a way of preserving them from the effects of the alkaloid, and advises them to put into the tube of the pipe or cigar-holder a little ball of cotton, impregnated with citric and tannic acids. As the smoke passes through the cotton, it will deposit the nicotine therein, in the shape of tannate and citrate. M. Melsens has made very ingenious experiments which go a very great way to prove that he is perfectly correct.—*Lancet.*

